# Beyond the Image: Advanced Ultrasound from Visualization to Quantitative and Precision Diagnosis

**DOI:** 10.3390/diagnostics16203284

**Published:** 2026-10-10

**Authors:** Maria Mezher, Laith R. Sultan

**Affiliations:** Department of Radiology, Children’s Hospital of Philadelphia, Philadelphia, PA 19104, USA

## 1. Introduction

Ultrasound has always excelled at showing us what is there. Its next chapter is increasingly about telling us what tissue is doing, how it is changing, and why those changes matter.

Advances in microvascular imaging, elastography, quantitative ultrasound, image post-processing, radiomics, and computational analysis are expanding ultrasound beyond conventional anatomical assessment. These techniques offer opportunities to characterize perfusion, tissue mechanics, acoustic properties, spatial heterogeneity, and dynamic behavior—information that may not be readily apparent by visual interpretation alone.

Importantly, this evolution builds on rather than replaces the qualities that have long made ultrasound distinctive: real-time imaging, portability, relative affordability, absence of ionizing radiation, dynamic assessment, and direct interaction with the patient. The field is therefore moving along an important continuum: from seeing to measuring, from measuring to understanding, and ultimately from understanding to guiding care.

The contributions to this Special Issue, “Advanced Ultrasound Techniques in Diagnosis”, provide a broad snapshot of this transition. Although they span different organ systems, patient populations, and technological approaches, they converge around several themes: visualization of previously inaccessible physiology, development of quantitative biomarkers, extraction of information beyond conventional visual assessment, and translation of imaging findings into clinically meaningful information.

## 2. Seeing What Was Previously Unseen

One of the most evident advances is the increasing ability of ultrasound to interrogate microvascular physiology.

Baş and Filiz evaluated ovarian stromal microvascularity in women with polycystic ovarian morphology using advanced microvascular Doppler imaging (contribution 1). Their study demonstrated the feasibility of quantifying stromal vascularity and identified associations with endocrine characteristics, illustrating how advanced Doppler techniques may provide physiological information beyond morphology alone.

The same investigators subsequently explored microvascular imaging in hepatic hemangiomas (contribution 2). Microvascular signals were detectable in lesions in which conventional color and power Doppler showed no flow, while lesion depth influenced signal detection. The study therefore illustrates both sides of technological progress: improved sensitivity can reveal previously inaccessible information, but acoustic and technical constraints remain relevant.

Rossmann et al. extended microvascular imaging beyond lesion characterization by studying median nerve perfusion during experimentally induced arterial occlusion (contribution 3). This application illustrates a broader transition from static imaging toward the assessment of physiologic response. As sensitivity to slow-flow vascularity improves, advanced Doppler methods may increasingly allow ultrasound to interrogate tissue perfusion rather than simply demonstrate the presence or absence of vessels.

Together, these studies suggest that the future of vascular ultrasound lies not only in showing blood flow more clearly, but in using vascular information as a marker of tissue biology and function.

## 3. From Appearance to Measurement

A second theme is the transition from qualitative tissue appearance toward quantitative tissue characterization.

Sultan et al. evaluated shear-wave elastography for carotid plaque characterization in a systematic review and meta-analysis (contribution 4). Differences in stiffness between symptomatic and asymptomatic plaques and the reported reproducibility of shear-wave measurements illustrate the potential of ultrasound to characterize mechanical properties that are not apparent from plaque morphology alone.

Santos and Reis examined this evolution in chronic liver disease, where elastography has become increasingly integrated into fibrosis assessment and longitudinal evaluation (contribution 5). Their review also highlights an important lesson that extends well beyond liver imaging: a numerical measurement is not necessarily an objective measurement. Patient factors, acquisition technique, equipment, quality criteria, and biological confounders may all influence quantitative ultrasound parameters.

This distinction will become increasingly important as the field develops more imaging biomarkers. The challenge is not simply to generate numbers, but to ensure that those numbers remain meaningful when the patient, operator, scanner, or institution changes.

Chuang et al. moved quantitative tissue characterization further by applying a scatteromics framework based on multimodal quantitative ultrasound envelope-statistics imaging to liver fibrosis assessment (contribution 6). Their approach uses properties of the ultrasound signal itself to construct quantitative descriptors related to acoustic scattering. Such approaches are particularly interesting because they may help establish a closer link between measurable ultrasound features and underlying tissue microstructure.

## 4. Beyond What the Eye Can See

The Special Issue also captures the increasing role of computational analysis in extracting information from routine ultrasound images.

De Leon-Benedetti et al. investigated grayscale ultrasound radiomics for differentiating acute kidney injury and chronic kidney disease in children (contribution 7). This pilot study demonstrated that texture features derived from conventional images could distinguish disease groups and support machine-learning classification. The broader implication is important: routine B-mode ultrasound may contain clinically relevant information that is present within the image but not readily accessible to human visual interpretation.

Mezher et al. examined this concept across pediatric imaging in their review of ultrasound radiomics (contribution 8). Current applications show promise across several organ systems, but the review also identifies substantial barriers to clinical translation, including small cohorts, retrospective designs, heterogeneous acquisition, segmentation variability, scanner dependence, and limited external validation.

These limitations are particularly relevant in ultrasound. Unlike modalities with more standardized image intensity scales, ultrasound appearance is strongly influenced by acquisition settings, transducer characteristics, image reconstruction, operator technique, acoustic windows, and post-processing. Computational analysis can potentially reduce subjectivity, but without adequate standardization it may also quantify technical variability rather than biology.

Not every advance, however, requires a complex machine-learning model. Hassan et al. demonstrated that post-processing ultrasound images using Fiji/ImageJ improved assessment of degenerative changes in uterine leiomyomas compared with conventional sonographic interpretation (contribution 9). This contribution underscores a useful principle: innovation does not always require entirely new imaging hardware. Sometimes, clinically useful information may already be present in routinely acquired images and simply require a better way of extracting or displaying it.

## 5. From Imaging Findings to Clinical Consequence

Advanced imaging becomes most valuable when it moves beyond technical performance and contributes to decisions that matter for patients.

Lee et al. prospectively compared ultrasound and MRI findings in women with placenta previa undergoing evaluation for placenta accreta spectrum and examined their relationship with perioperative hemorrhage (contribution 10). In the population studied, ultrasound impressions demonstrated an association with hemorrhagic outcomes. The significance of such work extends beyond the specific clinical setting: advanced imaging research should increasingly ask not only “Can we detect this?” but also “Does detecting it help us predict what happens next?”

The dynamic and interventional capabilities of ultrasound are illustrated by Yoon et al., who used high-resolution dynamic ultrasound to identify motion-dependent snapping of the superficial radial nerve in a patient with an atypical presentation of Wartenberg’s syndrome and subsequently performed ultrasound-guided hydrodissection (contribution 11). Even as ultrasound becomes increasingly quantitative and computational, this real-time ability remains one of its defining strengths. Few imaging modalities can integrate anatomy, motion, clinical examination, procedural guidance, and immediate reassessment within a single encounter.

## 6. What Comes Next?

Taken together, these contributions suggest that advanced ultrasound is moving toward a multiparametric future. Rather than relying on a single image or measurement, future examinations may combine anatomy, vascularity, perfusion, mechanics, acoustic properties, texture, and computational biomarkers according to the clinical question.

However, producing more parameters should not itself be considered progress. The real challenge is identifying which measurements are reproducible, biologically meaningful, and actionable.

## 7. Several Priorities Emerge

First, standardization must become part of innovation rather than something addressed after a promising result has been obtained. Acquisition protocols, scanner settings, transducer selection, image quality, segmentation, and post-processing can all affect quantitative measurements. Future studies should incorporate test–retest assessment, interobserver reproducibility, transparent acquisition reporting, and, where possible, cross-platform and multicenter evaluation.

Second, the field must progress from feasibility toward external validation. Many new ultrasound techniques appropriately begin in small, carefully selected cohorts. Translation, however, requires demonstrating that performance persists in more heterogeneous populations and across operators, equipment, and institutions.

Third, future work should place greater emphasis on biological interpretability. Computational methods can generate hundreds or thousands of imaging features, but statistical discrimination alone does not establish biological significance. Connecting quantitative ultrasound findings with pathology, tissue microstructure, physiology, molecular biomarkers, treatment response, and longitudinal outcomes may be more important than simply increasing model complexity.

Fourth, artificial intelligence should be explored not only as a mechanism for automated diagnosis but also as a means of addressing one of ultrasound’s oldest limitations: operator dependence. Future systems could potentially assist with anatomy recognition, standard-view identification, image-quality assessment, probe guidance, segmentation, automated measurements, and protocol adherence. In this context, AI would not simply interpret an examination after acquisition; it could help standardize how the examination is performed.

Fifth, areas that remain incompletely represented in the current collection may be equally informative in defining what comes next. Contrast-enhanced ultrasound, super-resolution imaging, AI-guided acquisition, multimodal integration, and automated quantitative analysis represent logical extensions of the themes explored in this Special Issue. Future research should examine how these technologies interact rather than evaluating each in isolation.

Perhaps most importantly, the next generation of advanced ultrasound studies should move beyond diagnostic accuracy alone.

A high area under the receiver operating characteristic curve is useful, but it does not by itself establish clinical value. Future investigations should increasingly determine whether an advanced ultrasound technique changes management, improves diagnostic confidence, predicts meaningful outcomes, reduces unnecessary testing or invasive procedures, improves workflow, or makes high-quality imaging more accessible.

That final point is particularly important. Ultrasound’s greatest advantage may ultimately be not only what it can measure, but where it can measure it: at the bedside, in the clinic, in the operating room, in emergency environments, and in settings where more resource-intensive imaging may not be readily available. Technological sophistication should preserve rather than diminish this accessibility.

## 8. From Better Images to Better Decisions

The papers assembled in this Special Issue demonstrate that the transformation of ultrasound is already underway.

Microvascular imaging is revealing flow that conventional techniques may not detect. Elastography and quantitative ultrasound are converting physical tissue properties into measurable biomarkers. Radiomics and computational analysis are uncovering patterns beyond visual interpretation. Image post-processing is extracting additional value from routine acquisitions. Dynamic ultrasound continues to connect diagnosis directly with physiology and intervention.

Yet the next major advance may not come from adding another isolated imaging parameter.

It may come from connecting the information we already know how to acquire—integrating anatomy with physiology, imaging phenotype with tissue biology, quantitative biomarkers with clinical context, and technological performance with patient outcomes.

In that sense, the future of ultrasound is not simply about producing better images.

It is about extracting better information from those images—and turning that information into better decisions for patients.

We sincerely thank all of the authors who contributed their research, experience, and perspectives to this Special Issue. We also thank the reviewers for their time and expertise and the editorial team of *Diagnostics* for their support throughout the development of this collection. It is our hope that the work presented here will stimulate continued collaboration among clinicians, imaging scientists, sonographers, engineers, and data scientists and contribute to the translation of advanced ultrasound technologies from promising innovations into robust and clinically meaningful tools.

